# Migraine incidence in 5 years: a population-based prospective longitudinal study in Turkey

**DOI:** 10.1186/s10194-015-0589-2

**Published:** 2015-12-03

**Authors:** Betul Baykan, Mustafa Ertas, Necdet Karlı, Derya Uluduz, Ugur Uygunoglu, Esme Ekizoglu, Elif Kocasoy Orhan, Sabahattin Saip, Mehmet Zarifoglu, Aksel Siva

**Affiliations:** Department of Neurology, Istanbul Faculty of Medicine, Istanbul University, Millet Cad, 34390 Capa/Istanbul, Turkey; Department of Neurology, School of Medicine, University of Uludag, Bursa, Turkey; Department of Neurology, Cerrahpasa School of Medicine, Istanbul University, Millet Cad, 34390 Capa/Istanbul, Turkey

**Keywords:** Migraine, Incidence, Headache epidemiology

## Abstract

**Background:**

The incidence of migraine has been investigated only in a few studies worldwide and it is not known in our country. We, therefore, aimed to estimate the migraine incidence in a previously accomplished population-based prevalence study sample of 5323 individuals in the year 2008.

**Methods:**

The former Turkish headache prevalence study has been completed as a nationwide, randomized, home-based study of face-to-face examination by physicians trained for headache diagnosis by using ICHD criteria. Five years after this study an optimized survey including 50 questions was performed to estimate the migraine incidence in migraine-free individuals in the previous study, with a 56.4 % responder rate. Two validation studies for this survey were performed prior and after the study each in 100 subjects by comparing the gold standard of expert diagnosis of headache, showing high rate of reliability (Crohnbach alpha: 0.911 and 0.706, respectively).

**Results:**

Migraine incidence was estimated as 2.38 % (2.98 % in women and 1.93 % in men) per year in 2563 migraine-free individuals; if the population at risk is defined as the group without any headaches, the migraine incidence decreased to 1.99 %. The chronic migraine (CM) incidence [without medication overuse (MOH)] was 0.066 % and that of MOH was 0.259 %. We found a significant burden of the disease on the occupational functionality as well as on social and family life, even in the early years of the migraine. The family history of headaches especially in the fathers could be useful to predict new cases of migraine, besides the well-known risk factor, diagnosis of depression, whereas income and education did not seem to relate to migraine onset.

**Conclusions:**

Our study with a large population-based nation-wide sample, using ICHD-II criteria, with structured headache interviews as well as blinded re-validation of the questionnaire diagnoses showed a 2.38 % incidence rate of migraine in Turkey, higher than most of the other previous reports; a finding which could be related to genetic factors and also to the methodological differences in the study designs. Moreover the incidence of CM was found to be 0.066 %.

## Background

Headache was highlighted as the third cause of disability worldwide and migraine is a frequent form of headache disorders causing remarkable disability during the headache attacks along with substantial social and financial burden to the patient as well as to the society [1, 2]. The prevalence of migraine for Western countries is established as nearly between 5 and 9 % for men and between 12 and 25 % for women [3, 4]. These figures are subject to change between countries and races; reported rates of migraine are lower in Asia and Africa than in Western countries. Prevalence studies have already demonstrated the magnitude of health impact caused by migraine but incidence studies needing further huge efforts may contribute to our understanding of migraine mechanisms towards the identification of important risk factors. The introduction of the International Classification of Headache Disorders (ICHD) have stimulated valuable prevalence studies of population-based data, however, there is only one population-based incidence study done in Germany with new ICHD-II edition so far [3–7].

There are still many methodological problems with respect to epidemiological studies about the migraine [3–7]. Unfortunately, migraine incidence studies are surprisingly scant due to its challenging aspects; as a result relatively little is known about its incidence. Besides being uncommon many of the migraine incidence studies carry some methodological concerns or confined to selected age groups such as children and adolescents [8–12].

Furthermore the estimated migraine incidence figures from these studies are quite variable. This variability may depend on methodological differences, on case definitions and study characteristics; many of the previous studies based on small samples of relatively few new-onset cases [3, 7]. Moreover Stewart et al. showed in a cross-sectional survey study with a large sample and broad age range that the cumulative lifetime risk of migraine is 2.5–3 times higher than prevalence, intriguingly [13].

We previously published a nation-wide population-based prevalence study in our country bridging Asia and Europe and the gold-standard of headache diagnosis established by face to face personal interview and examination by physicians were applied in this first study [14]. Our current aim is to estimate the migraine incidence in this previously well-investigated population sample, for the first time in Turkey. Our secondary aim was to assess the associated risk factors for migraine development in these series.

## Methods

### Two steps of the incidence study

1) The first step of this study, namely Turkish headache prevalence study was completed in 2008 on 5323 households aged between 18 to 65 years. This former study was designed as a nationwide, randomized, home-based study of face-to-face examination by general practitioners specifically educated for headache diagnosis by using ICHD-II criteria and published in 2012 [14]. In 2013, five years after this prevalence study, a second study was planned to be done with the same subjects of the first study. We aimed to contact all individuals who were migraine-free in 2008 to calculate the incidence of migraine in the general Turkish population.

2) The second part of study was designed in 2 sessions by headache experts as a telephone survey of all included subjects of the first study. For this telephone survey, a new questionnaire with 50 questions consisting of both diagnostic and investigational points was prepared. The survey was first optimized for convenient use of time and intelligibility of the questionnaire after first 10 telephone interviews. Trained call center employees did the telephone calls, asked the questions of the questionnaire and recorded the answers first by re-taking the consent of the called subject after reminding his/her first contribution to the study 5 years ago. For the validation of current telephone survey, two different validation studies were performed in two phases; first one was the “pre-study validation” and second one was the “post-study validation”. Ethical committee approved the study protocol.

### Pre-study validation

In the beginning of the study, 100 subjects living in two cities were randomly selected among 5323 subjects of the first study. First the call center employees had called them and implemented the questionnaire as planned. Then, the same subjects were invited to the headache centers of the university hospitals (in the metropolis of Istanbul and Bursa) for a face-to-face interview by the headache experts to establish the gold standard of “clinical headache diagnosis”. Of 100 subjects, 49 have accepted the invitation and received headache diagnoses by experts. The interobserver reliability analysis of headache diagnoses between the telephone interview and the headache specialist examination revealed that the questionnaire was excellently reliable (Crohnbach alpha: 0.911).

### Telephone survey

Fifty-item questionnaire intended to be implemented in a total of 5323 households could be administered in 3001 subjects, with a 56.4 % responder rate. Of 3001 subjects, 2563 [currently aged between 23 and 71 years (mean ± standard deviation; 41.7 ± 12)] were migraine-free at the time of the first prevalence study in the year of 2008 and they were used in the calculation of migraine incidence for the purpose of the current study.

### Post-study validation

In the end of the telephone survey study, another randomly selected sample of 100 subjects living in the same cities were invited to the university hospitals for a face to face interview with the headache specialists to make a clinical headache diagnosis. Of 100 subjects, 73 accepted to come to the visit. The reliability analysis of headache diagnoses between questionnaire by telephone call and the headache diagnoses by the headache specialist revealed that the questionnaire was again strongly reliable (Crohnbach alpha: 0.706).

### Secondary aims

Risk factors for migraine development were calculated based on the status of migraine-free individuals in 2008, comparing the subgroups with and without definite migraine in 2013. Incidence of chronic migraine (CM) and medication overuse headache (MOH) was also estimated in the study population excluding only patients with CM or MOH diagnosis in 2008. The impact of migraine on the patients’ life and their headache burden were also investigated based on questionnaire. Furthermore, medical consultation for headache of the patients in 2008 and of those in 2013 (without any headache in 2008) has been explored.

### Statistical analysis

SPSS 15.0 program was used for statistical analysis. Descriptive statistics were applied for the relevant parameters. Reliability analysis (Crohnbach alpha) was done between the telephone survey and expert diagnosis, before and after the survey. Chi-square test and t test were used for the group comparisons, where appropriate.

## Results

**Fig. 1:**
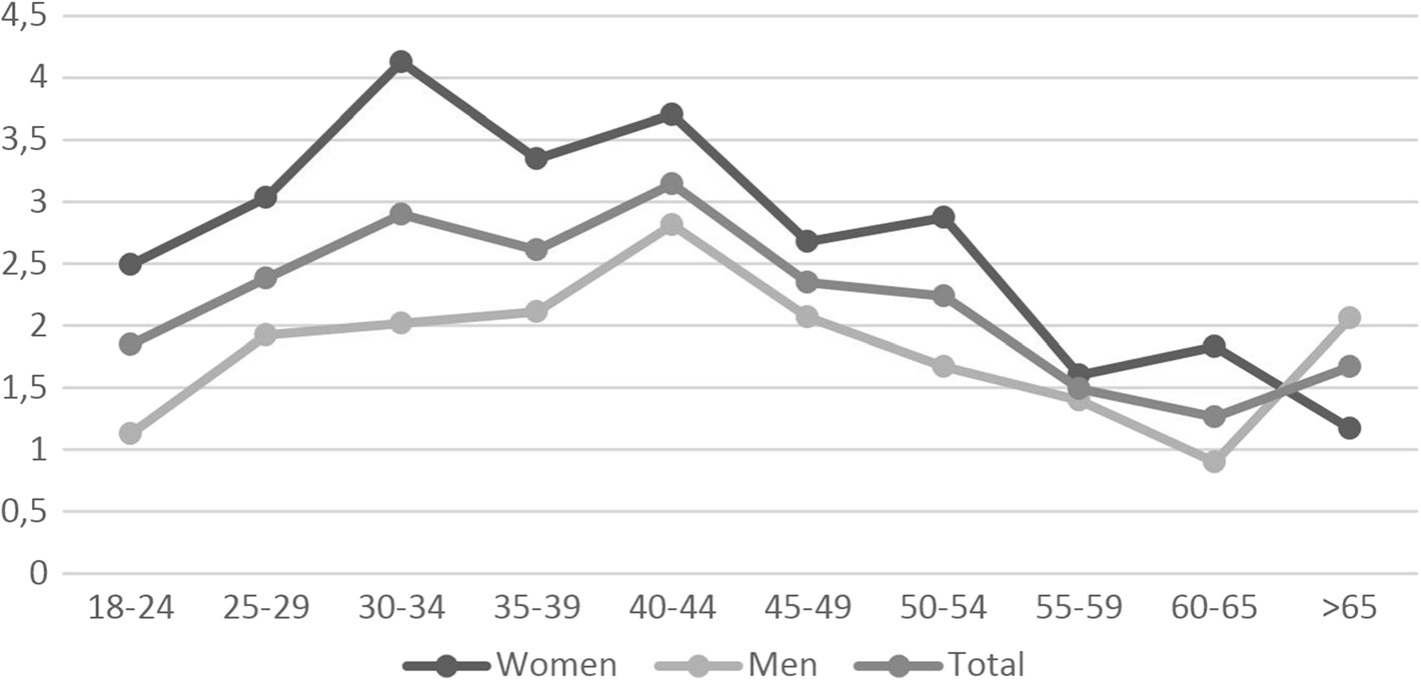
The annual incidence rates in age groups

Migraine incidence is estimated as 2.38 % per year, from 314 new incident cases in 2563 subjects (the migraine-free individuals in the year 2008 defined as subjects who were not diagnosed as definite migraine according to ICHD criteria) in the following 5.15 years. The incidence is 2.98 % per year in women and 1.93 % per year in men. Figure 1 shows the annual incidence rates in age groups, both for women and men. If the population at risk is defined as the group without any headaches in the year 2008 [1839 participants without any headache (747 females, 1092 males)], the calculated migraine incidence falls to 1.99 % (2.44 % females, 1.67 % males), whereas headache incidence is 8.67 % per year (9.91 % females, 7.83 % males) (Table 1).

**Table 1:** Migraine incidence and prevalence estimates in different age groups

	Migraine incidence			Migraine prevalence			OR (95 % CI)
	(excluding old migraineurs)			(including old migraineurs)			
	per year (%) *n* = 2563			(%) *n* = 3001			
	Women	Men	Total	Women	Men	Total	
Age groups (years) 18–24	2.49	1.13	1.85	15.7	8.3	12.4	0.487 (0.177–1.340)
25–29	3.03	1.91	2.38	20.9	10.4	15.3	0.436 (0.251–0.760)
30–34	4.12	2.02	2.90	26.6	12.5	18.7	0.392 (0.258–0.618)
35–39	3.35	2.10	2.61	22.6	15.2	18.4	0.612 (0.370–1.012)
40–44	3.70	2.81	3.14	25.5	16.7	20.7	0.585 (0.345–0.991)
45–49	2.68	2.07	2.35	25.5	15.5	20.7	0.535 (0.316–0.907)
50–54	2.87	1.67	2.24	20.0	9.3	14.7	0.409 (0.203–0.825)
55–59	1.60	1.40	1.49	16.8	10.7	13.7	0.596 (0.279–1.270)
60–65	1.83	0.90	1.26	13.8	6.7	9.7	0.444 (0.150–1.318)
65+	1.17	2.06	1.67	10.0	10.6	10.3	1.068 (0.338–3.376)
18–70	2.98	1.93	2.38	21.6	12.4	16.7	0.512 (0.421–0.623)

The new migraine prevalence, which was 16.4 % in this cohort in 2008, is estimated as 16.7 % in 3001 persons, aged between 23 and 70 years, who answered the questionnaire in 2013.

Moreover, CM incidence (without medication overuse) was 0.066 %; and the incidence of MOH was 0.259 % in 2928 subjects excluding only CM or MOH (73 patients), and including episodic migraineurs in the year 2008. Among the 314 new incident cases with definite migraine, the rate of CM was 0.56 % (0.8 % in males and 0.35 % in females) and that of MOH was 1.05 %. Tables 2 and 3 summarizes data of CM and MOH in Turkish population. The most overused analgesic drugs are nonsteroidal analgesic drugs (65.6 %) followed by simple analgesics (34.4 %), ergotamins (18.8 %) and triptans (15.6 %) in new incident 32 patients. Headache characteristics and burden of patients with new incident migraine were given in the Table 4.

**Table 2:** Chronic migraine (CM) incidence and prevalence values*

Age groups (years)	CM incidence			CM prevalence			OR (95 % CI)
	(%) *n* = 2928			(%) *n* = 502			
	Women	Men	Total	Women	Men	Total	
18–24	0.000	0.000	0.000	0.0	0.0	0.0	
25–29	0.000	0.000	0.000	0.0	4.3	1.6	1.045 (0.958–1.141)
30–34	0.085	0.000	0.038	1.6	1.0	1.0	0.984 (0.952–1.016)
35–39	0.114	0.000	0.049	2.5	0.0	1.3	0.975 (0.928–1.025)
40–44	0.131	0.106	0.117	2.5	6.5	4.2	2.690 (0.233–31.109)
45–49	0.222	0.114	0.169	4.3	7.4	5.4	1.800 (0.239–13.569)
50–54	0.140	0.0000	0.070	3.4	0.0	2.4	0.966 (0.901–1.034)
55–59	0.000	0.971	0.085	0.0	7.7	3.1	1.083 (0.926–1.267)
60–65	0.000	0.000	0.000	0.0	0.0	0.0	
65+	0.329	0.000	0.157	16.7	14.3	15.4	0.833 (0.041–16.994)

*CM (without medication overuse) incidence values among the population without CM or medication overuse headache (MOH) diagnosis in 2008 (including old episodic migraineurs) and CM prevalence values in patients with definite migraine (including old migraineurs) in 2013 study

**Table 3:** Medication overuse headache (MOH) incidence and prevalence values*

Age groups (years)	MOH incidence			MOH prevalence			OR (95 % CI)
	(%) *n* = 2928			(%) *n* = 502			
	Women	Men	Total	Women	Men	Total	
18–24	0.452	0.000	0.246	14.3	0.0	10.0	0.857 (0.692–1.062)
25–29	0.313	0.177	0.239	5.0	4.3	4.8	0.864 (0.074–10.081)
30–34	0.511	0.000	0.227	6.6	0.0	4.1	0.934 (0.874–0.999)
35–39	0.454	0.260	0.344	5.0	8.6	6.7	1.781 (0.280–11.328)
40–44	0.656	0.106	0.352	10.0	6.5	8.5	0.621 (0.106–3.631)
45–49	0.111	0.114	0.113	4.3	3.7	4.1	0.865 (0.075–10.014)
50–54	0.279	0.142	0.211	6.9	7.7	7.1	1.125 (0.093–13.636)
55–59	0.890	0.162	0.509	26.3	0.0	15.6	0.737 (0.563–0.064)
60–65	0.000	0.000	0.000	0.0	0.0	0.0	
65+	0.000	0.597	0.313	0.0	14.3	7.7	1.167 (0.862–1.579)

*MOH incidence values among the population without chronic migraine (CM) or MOH diagnosis in 2008 (including old episodic migraineurs) and MOH prevalence values in patients with definite migraine (including old migraineurs) in 2013 study

**Table 4:** Headache characteristics and burden of patients with new incident migraine

	Women	Men	Total
	*n* = 168	*n* = 146	*n* = 314
Headache frequency per month mean (± SD)	5.5 ± 5	5.2 ± 5	5.3 ± 5
Headache days per month mean (± SD)	5.9 ± 5	5.4 ± 6	5.7 ± 6
Headache duration (hours) without medication mean (± SD)	27.0 ± 95	20.9 ± 84	24.2 ± 90
Headache severity (0–3 scale) mean (± SD)	2.06 ± 0.7	2.03 ± 0.6	2.05 ± 0.6
Self Assessment of Poor Quality of Life (%)			
Usually	34	32	33
Sometimes	45	47	46
Never	21	21	21
Economic loss due to migraine (%)			
Usually	20	15	18
Sometimes	33	38	35
Never	47	47	47
Poor relations with family (%)			
Usually	32	22	28
Sometimes	35	40	37
Never	33	38	35
Poor relations with friends (%)			
Usually	32	24	28
Sometimes	31	36	33
Never	37	40	39
Loss of work time (workers) (%)	(*n* = 65)	(*n* = 119)	(*n* = 184)
Yes	34	27	29
No	66	73	71
Loss of school time (students) (%)	(*n* = 12)	(*n* = 8)	(*n* = 20)
Yes	42	50	45
No	58	50	55

*SD* Standard deviation

**Fig. 2:**
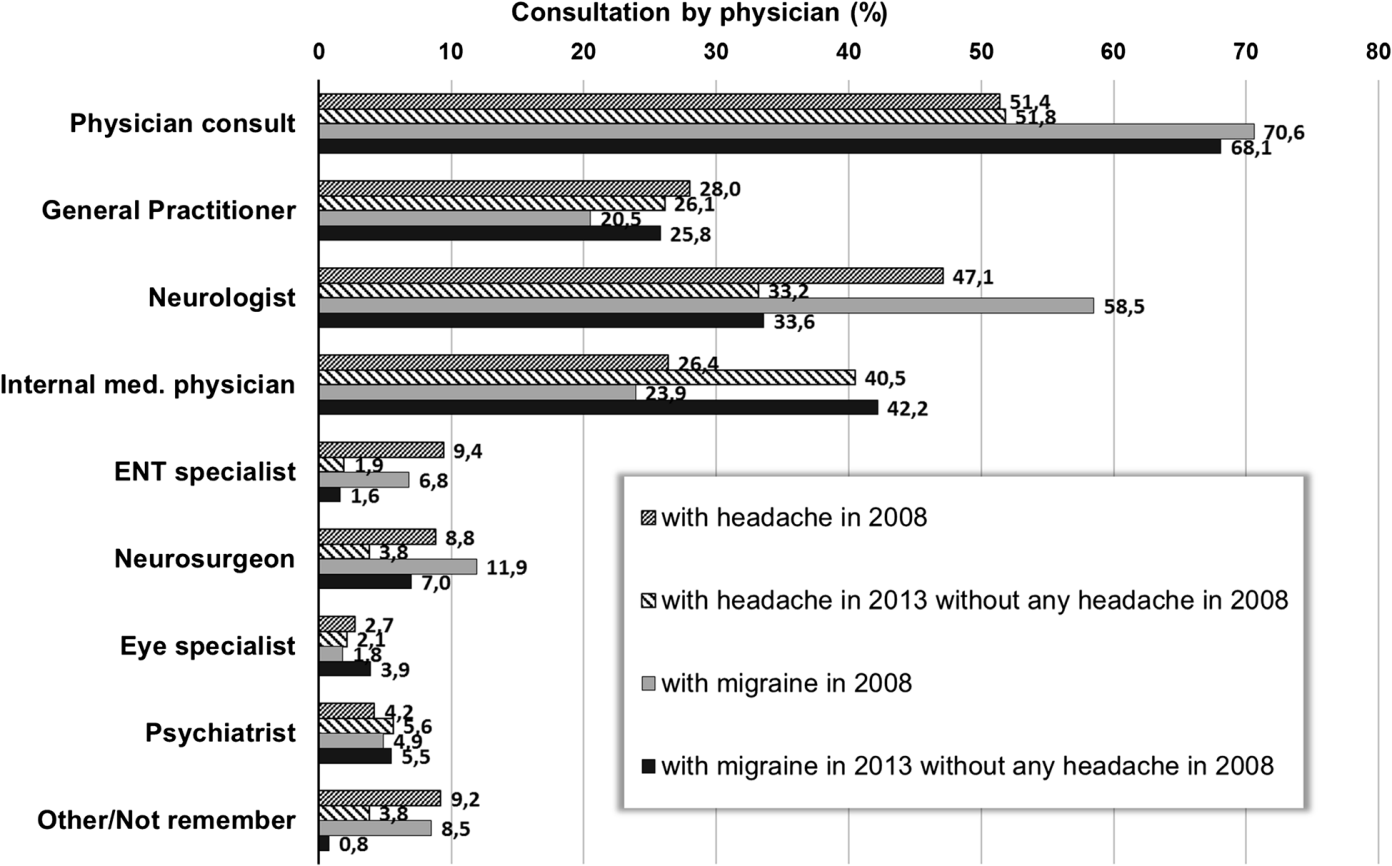
Consulted type of physician for headache and with definite migraine in 2008 and in 2013

We also compared some possible baseline predictive factors like education, income status, diagnosis of depression and family history of migraine between new incident cases of definite migraine and migraine-free individuals in the years 2008 through 2013 (Table 5). Moreover, the consulted physicians’ specialty in the years 2008 and 2013 were both shown in the Fig. 2.

**Table 5:** The comparison of some baseline risk factors between new incident cases of definite migraine and migraine-free individuals*

Assessed factor	New-onset migraineurs	Migraine-free individuals	*P*	Odd Ratio (95 % CI)
	(n:314)	(n: 2249)		
Monthly income				
more than 1300USD	95 (30.3 %)	621 (27.6 %)	NS	1.137 (0.878–1.471)
less than 1300USD	219 (69.7 %)	1628 (72.3 %)		
Diagnosis and treatment for depression**			0.010	
yes	58 (18.8 %)	296 (13.4 %)		1.503 (1.102–2.051)
no	250 (81.2 %)	1918 (86.6 %)		
Headache in family				
yes	172 (54.8 %)	1069 (47.5 %)	0.016	1.337 (1.055–1.695)
no	142 (45.2 %)	1180 (52.5 %)		
Mother with headache				
yes	93 (29.6 %)	631 (28.1 %)	NS	1.079 (0.833–1.398)
no	221 (70.4 %)	1618 (71.9 %)		
Father with headache				
yes	54 (17.2 %)	243 (10.8 %)	0.001	1.715 (1.243–2.366)
no	260 (82.8 %)	2006 (89.2 %)		
Education				
more than 8 years	228 (72.6 %)	1598 (71.1 %)	NS	1.080 (0.829–1.407)
8 years or less	86 (27.4 %)	651 (28.9 %)		

*these comparison groups included only migraine-free individuals in the baseline (year 2008). ** Six migraineurs and 35 migraine-free individuals do not remember any history of depression

*Ns*:not significant with Pearson’s chi-square test (two-sided)

## Discussion

Our migraine incidence study complements the previous nationwide prevalence study and showed that migraine incidence is 2.38 % (2.98 % for women and 1.93 % for men) per year in Turkey in the last 5 years. Moreover, the incidence of CM and MOH were investigated in CM and MOH-free subjects and found to be 0.066 and 0.259 %, respectively. Our results also showed the significant burden of migraine on the occupational functionality as well as on social and family life, even in the early years of the migraine. We also noted that the history of headaches in the fathers could be useful to predict new cases of migraine, besides the well-known risk factor, diagnosis of depression.

### Comparison of our findings with other incidence studies

Due to the challenging aspects, only a handful of previous studies have been published on the incidence of migraine so far. Some retrospective reports [15, 16] have estimated the incidence of migraine from recalled age at onset in cross-sectional studies with telephone interviews only and therefore have limitations related to this retrospective design, like recall bias. The retrospective studies and another study [17] which investigated medical record system, reported incidence rates about 1.5–2 per 1000 person-years for men, and about 3–6 per 1000 person-years for women, all seem rather low in comparison to our data. The migraine incidence rate was found as 3.69 cases per 1000 person-years, in a study based on the general practitioners’ records in UK [18]. On the other hand Breslau et al. estimated the incidence of migraine per 1,000 person-years as 5.0 for males and 22.0 for females in United States, in a follow-up study of adults aged 21–30 years from a large health maintenance organization; where they also aimed to assess the bidirectional influences between major depression and migraine. Their study group was smaller than ours and therefore they likely underestimated the incidence of migraine because they included subjects from a very narrow age range [19, 20]. Swartz et al. did not really assess migraine at baseline but screened the population at-risk and found similar figures [21]. Moreover in US, migraine incidence data was estimated using reconstructed cohort methods [13], the incidence (1.8 %) peaked in women aged 20 to 24 years, whereas they found the peak in 15- to 19-year-old men (0.6 %). An important population-based Danish study [9] found a rate of 0.8 % per year (male:female ratio, 1:6; among 740 individuals examined in 1989, 673 were eligible in 2001 and 549 participated only; of the 453 subjects ranging in age from 25 to 64 years who did not have migraine in 1989, 42 developed migraine during the study period) in the general population of a single city (Copenhagen) following a previous study after 12 years. They used ICHD criteria and the interviews were done by a physician, either by personal examination or by telephone interviews. Their initial modest-sized cohort was restricted to adults aged 25–64 years, thus restricting them to estimate incidence rates in younger age groups and the annual incidence in females was 1.5 % and in males 0.3 %. They reported that migraine incidence rates declined with age, was highest in the 25 to 34 years of age group in both women and men and was lowest for both genders in the 55 to 64 years of age group; somewhat similar to our findings, except that we found the highest rate in 40–44 age group in men. Thus the differences in study populations (e.g. age, geographical area) may as well have an effect on the reported differences, besides methodological aspects.

Our study investigated a larger population sample after 5 years in 21 cities of Turkey with ICHD-II and our incidence figure of 2.38 % is higher than most of the other previous reports, which used ICHD-I. Also the migraine prevalence rates in Turkey have been investigated 3 times [in 1998, 2007 and 2008 [14, 22, 23]] and now in 2012 were more or less the same (between 16.4 and 16.7 %) which were again higher than most of the reported prevalence rates, supporting this high migraine incidence in our country. Genetic factors may contribute to these higher prevalence and incidence rates in Turkey, but it is possible that these high rates may as well relate to study methodology.

Furthermore, a recent carefully designed German study using ICHD-II has drawn attention to the definition of “people at risk for migraine” and suggested that different definitions of the population at risk limits the comparability of the reported incidence estimates among the published prospective studies on the migraine incidence. They found that the incidence of migraine ranged between 0 and 3.3 % in three groups of participants a) without any headache b) without definite and probable migraine and c) without definite migraine, probable migraine and definite TTH at baseline [5]. In our study, if the population at risk was defined as the group without any headaches in the year 2008, the calculated migraine incidence declines to 1.99 %.

### Epidemiology of chronic migraine

CM has been a debatable definition in headache classification and was therefore mostly neglected in epidemiological studies or grouped as chronic daily headache (CDH) with or without MOH [1]. The average cross-study estimate of the prevalence of CM was 0.5 % with a range from 0.2 to 2.7 % with a possible bias from the application of different diagnostic criteria and definitions across studies [7, 24–26]. When patients with MOH were included, then the estimated global prevalence of CM ranged between 1 and 5 % [27–31].

In an interesting study, Bigal et al. showed that about 2.5 % of patients with episodic migraine progressed to CM over a one year period [32] by using Silberstein-Lipton criteria [33] treating MOH as a subset of patients within the CM group. We estimated the CM incidence (without medication overuse) as 0.066 % according to ICHD-II criteria and the rate of CM was 0.56 % in new incident patients diagnosed with definite migraine. To our knowledge, this is the first population based study presenting data on the incidence of CM. Furthermore, female sex is reported as a risk factor for CM in a recent study [34].

ICHD criteria specifically exclude MOH from the CM definition. A large prospective study from Norway reported that the MOH incidence was 0.72 per 1000 persons-years [35], which was higher than the incidence rate found in the current study. There is evidence for an association between the chronicity of headache and medication overuse [36, 37], but the causal relationship remains uncertain. Nearly one third of our patients with chronic headache did not have medication overuse similar to previous reports [38] which may suggest that the genetic factors might play a role in the development of headache chronification. The transition to CM may also be influenced by environmental factors such as lifestyle, awareness, education, detrimental life events, comorbid conditions besides personal genetic pattern [39]. On the other hand, Lipton et al. reported that inadequate treatment was a risk factor for new onset CM [40] and found that the greatest barrier for an appropriate treatment occurred at the level of consulting a health care professional [41]. Our findings revealed that 50–70 % of patients consulted a physician for headache but the new incident cases consulted more often an internal medicine specialist than a neurologist in our country. Thus, it seems that headache patients are less likely seen by a neurologist in the early years of their disease and this could also have an impact on the high CM incidence. Increasing awareness about the need for consultation by a neurologist, specifically trained for headache may help to prevent chronification of migraine.

### The burden of migraineurs

The individual impact of migraine related to the duration, severity and frequency of the attack is well-known, but the changes during years are not well investigated. However, migraine may also have cumulative impact over time. Many studies in the United Kingdom and the United States reported substantial impact and burden of migraine on family life, social relationships, like postponing their household work (85 %) canceling family and social activities (45 %) etc. [42–45]. Our study is unique to report the burden of headache particularly in new-onset migraineurs and confirmed that migraine can lead to disruption of work, family and social life and affect quality of life, even in the early stages of the disease.

### Speculations on predictors of migraine onset

Despite clear evidence of genetic contribution to migraine onset, modifying or triggering effects of some environmental factors such as education level, socio-economic status, and premorbid depression are also debated in the relevant reports.

- A higher prevalence of migraine was observed in clinical series of individuals having a higher level of education in some studies, whereas some other studies reported just the opposite [3, 46, 47] . Our current study did not show any effect of education on the migraine onset in migraine-free individuals, whereas in the same population, we had previously found lower migraine prevalence in those with a lower educational status than those with a high educational status [14].
- Some population-based studies have found that migraine prevalence is inversely related to income in the US [46, 48, 49]. On the other hand, no such correlation was found in studies conducted in Europe [50–53], nor in two other studies from North America [43, 54], similar to our results. However, in a recent study we showed that migraine prevalence was higher (26.4 %) among women with a lower income (less than 1,300 US$ monthly) than the ones with a higher income (20.3 %), while in men, it was the same in the ones with lower income (8.5 %) and the ones with higher income (8.5 %) [14].
- Many studies already pointed out that family history of migraine is especially important in migraine prediction and persistence [55, 56]. Our results showed that history of headache in the father is a more important predictor for new-onset cases for migraine. It could be wisely speculated that headache history in the mother is very frequent in the general population and therefore, the genetic tendency from paternal side may be more important for the migraine onset.
- Association of migraine with depression has also been well established in population based studies besides clinical samples [21, 57]. Our study supplied evidence that migraine-free individuals who received physician diagnosis and treatment for depression have significantly increased rates of new incident migraine when compared to those without depression history. Taken together with the increased family history, it is reasonable to conclude that this comorbidity may be based on a shared genetic background [58, 59].

### Weaknesses and strengths of our study

The current gold standard of headache diagnosis is personal interview and examination by an experienced physician; thus our initial study has fulfilled this standard. However, this is a rather expensive approach and in the second part of the incidence study we applied telephone interviews by trained staff according to ICHD-II criteria, which was reported to be acceptable for screening purposes [3]. Furthermore, this telephone questionnaire has been meticulously validated two times taking the expert diagnosis as the gold standard, which increased the reliability of our results. There are relatively few studies using validation of their methods in a sample after interview and examination by a neurologist or headache expert. [3, 60, 61] We have a moderate participation rate of 56.4 % for the telephone survey which could represent a potential selection bias. This rate is indeed not very low when the 5 years interval was taken into account and when compared to other similar studies. But subjects with headache (and even more subjects with a recent-onset headache) might be eager to participate in the headache interview when compared to headache-free individuals. Moreover it should be emphasized that our study underestimated migraine incidence in the 18–24 age group, since the original population in 2008 was aged over 18 years.

It has been well-known that there is overlap in symptomatology between headache subtypes and multiple headache types often coexist in the same individual; for example great majority of migraineurs also have tension type headache (TTH) creating diagnostic confusion for many studies [7]. Therefore we only investigated the migraine incidence to obtain rather solid results and mainly focused on patients strictly diagnosed with migraine according to the ICHD-II, which is currently most elaborate and comprehensive system for classifying migraine.

The strengths of our study were the representative sampling of a population-based nation-wide study accomplished by trained physicians, use of ICHD-II criteria, and the structured headache interviews, blinded re-evaluation of the previous diagnoses. The validity and reliability of telephone interviews were found to have very good agreement with the clinical interviews in studies assessing the telephone interviews by comparison with the “gold standard” which was accepted as clinical interviews by experts [60].

## Conclusions

Our study showed a 2.38 % incidence rate of migraine in Turkey, higher than most of the other previous reports; a finding which could be related to genetic factors and also to the methodological differences in the study designs. Moreover, the incidence of CM was found to be 0.066 % and that of MOH was 0.259 %.
